# Supplementary figures and images for: Activation of mTORC1 and c-Jun by Prohibitin1 loss in Schwann cells may link mitochondrial dysfunction to demyelination
Source: eLife. 2021 Sep 14;10:e66278. doi: 10.7554/eLife.66278 (PMC8478418; doi:10.7554/eLife.66278)

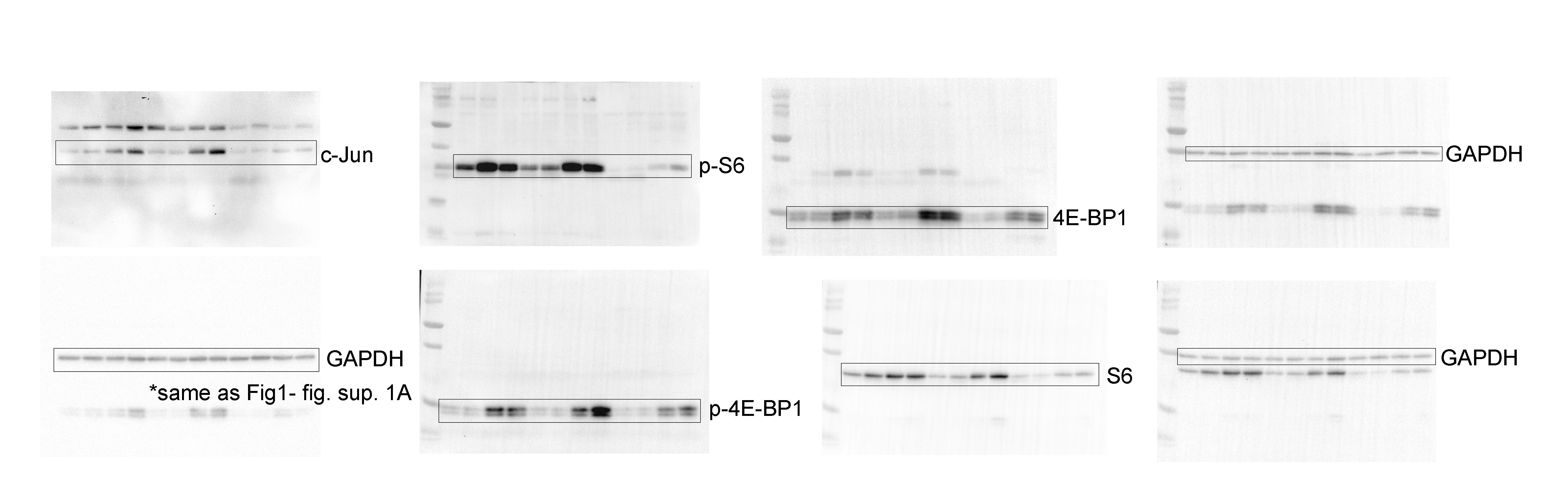

Supplement: Figure 1—source data 1. [file elife-66278-fig1-data1.zip › Figure 1 - source data 1/Annotated uncropped WB.jpg]

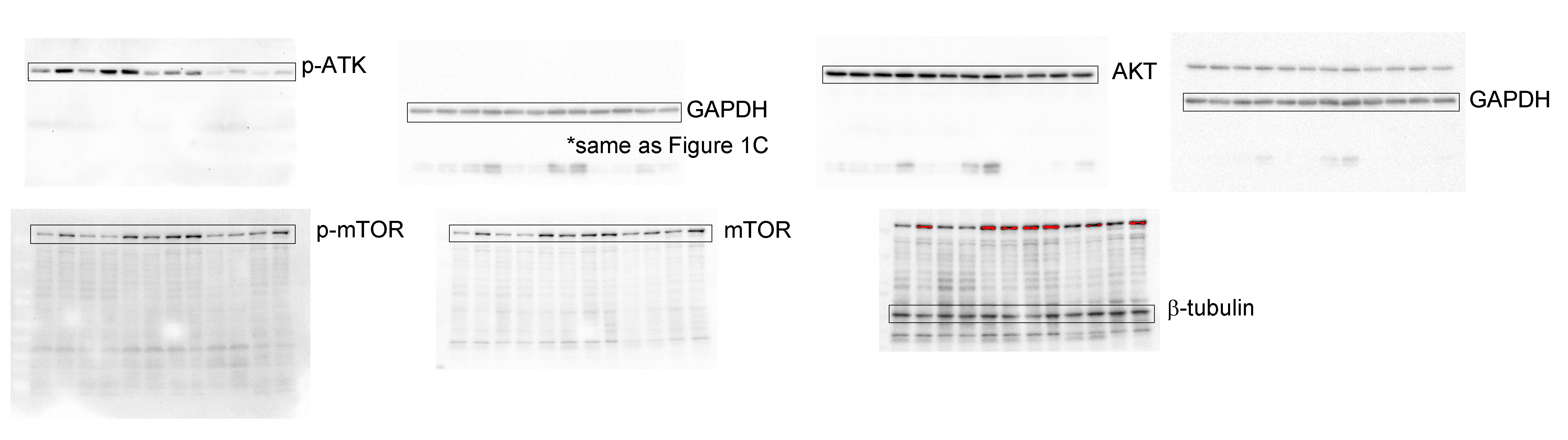

Supplement: Figure 1—figure supplement 1—source data 1. [file elife-66278-fig1-figsupp1-data1.zip › Figure 1 - figure supplement 1 - source data 1/Annotated uncropped WB.jpg]

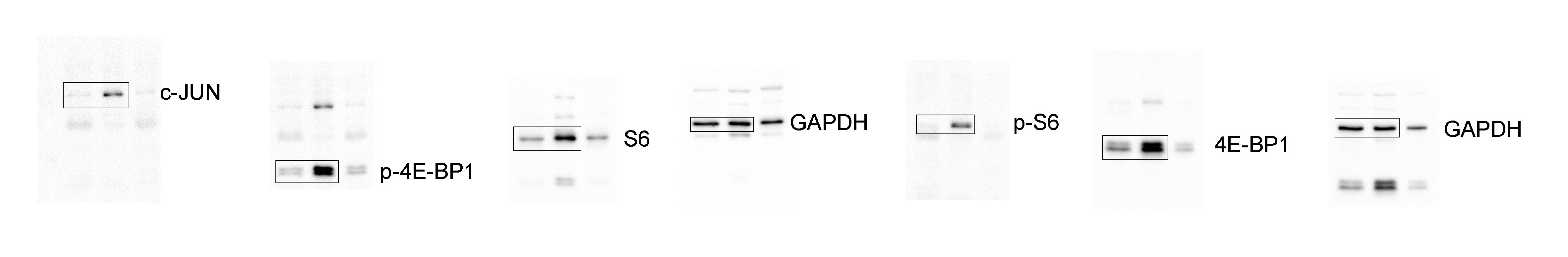

Supplement: Figure 1—figure supplement 2—source data 1. [file elife-66278-fig1-figsupp2-data1.zip › Figure 1 - figure supplement 2 - source data 1/Annotated uncropped WB.jpg]

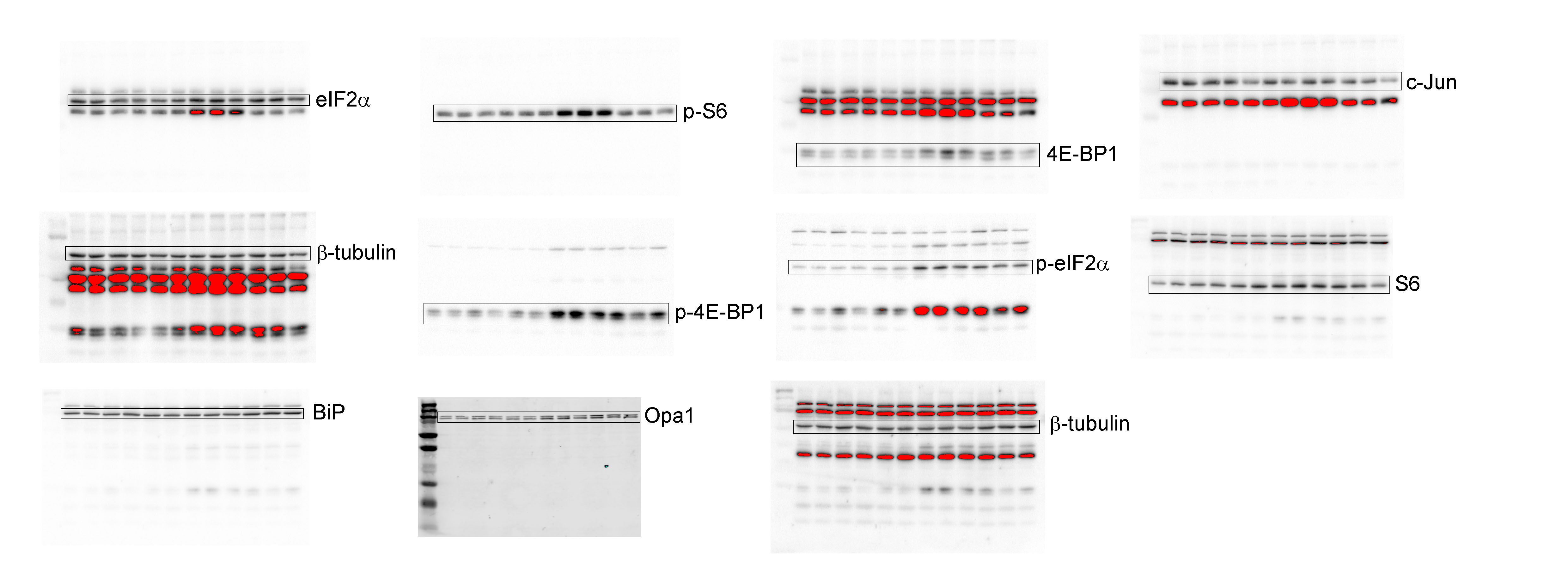

Supplement: Figure 2—source data 1. [file elife-66278-fig2-data1.zip › Figure 2 - source data 1/Annotated uncropped WB.jpg]

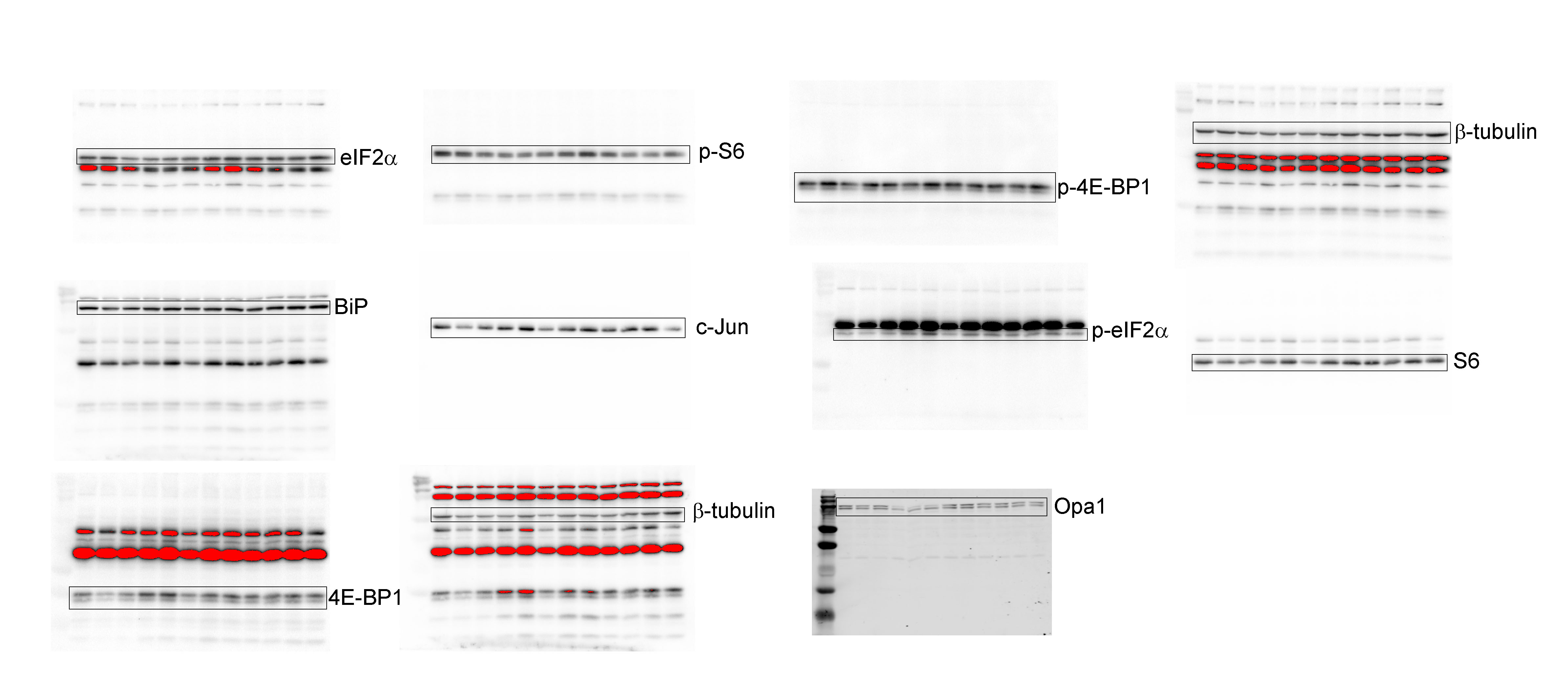

Supplement: Figure 2—figure supplement 1—source data 1. [file elife-66278-fig2-figsupp1-data1.zip › Figure 2 - figure supplement 1 - source data 1/Annotated uncropped WB.jpg]

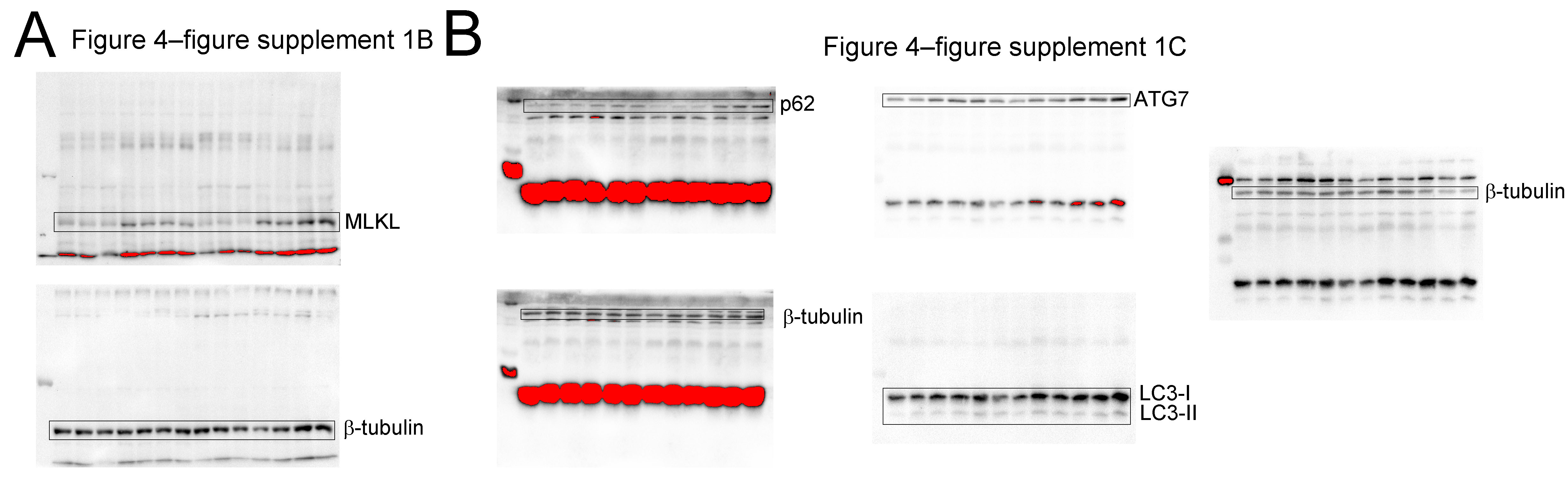

Supplement: Figure 4—figure supplement 1—source data 1. [file elife-66278-fig4-figsupp1-data1.zip › Figure 4 - figure supplement 1 - source data 1/Annotated uncropped WB.jpg]

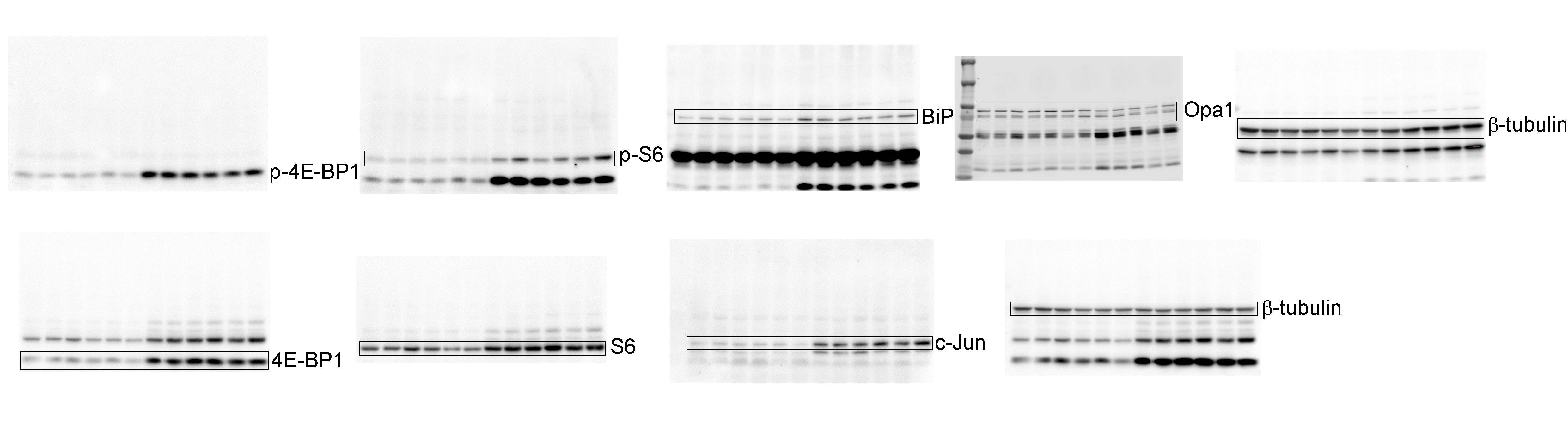

Supplement: Figure 5—source data 1. [file elife-66278-fig5-data1.zip › Figure 5 - source data 1/Annotated uncropped WB.jpg]

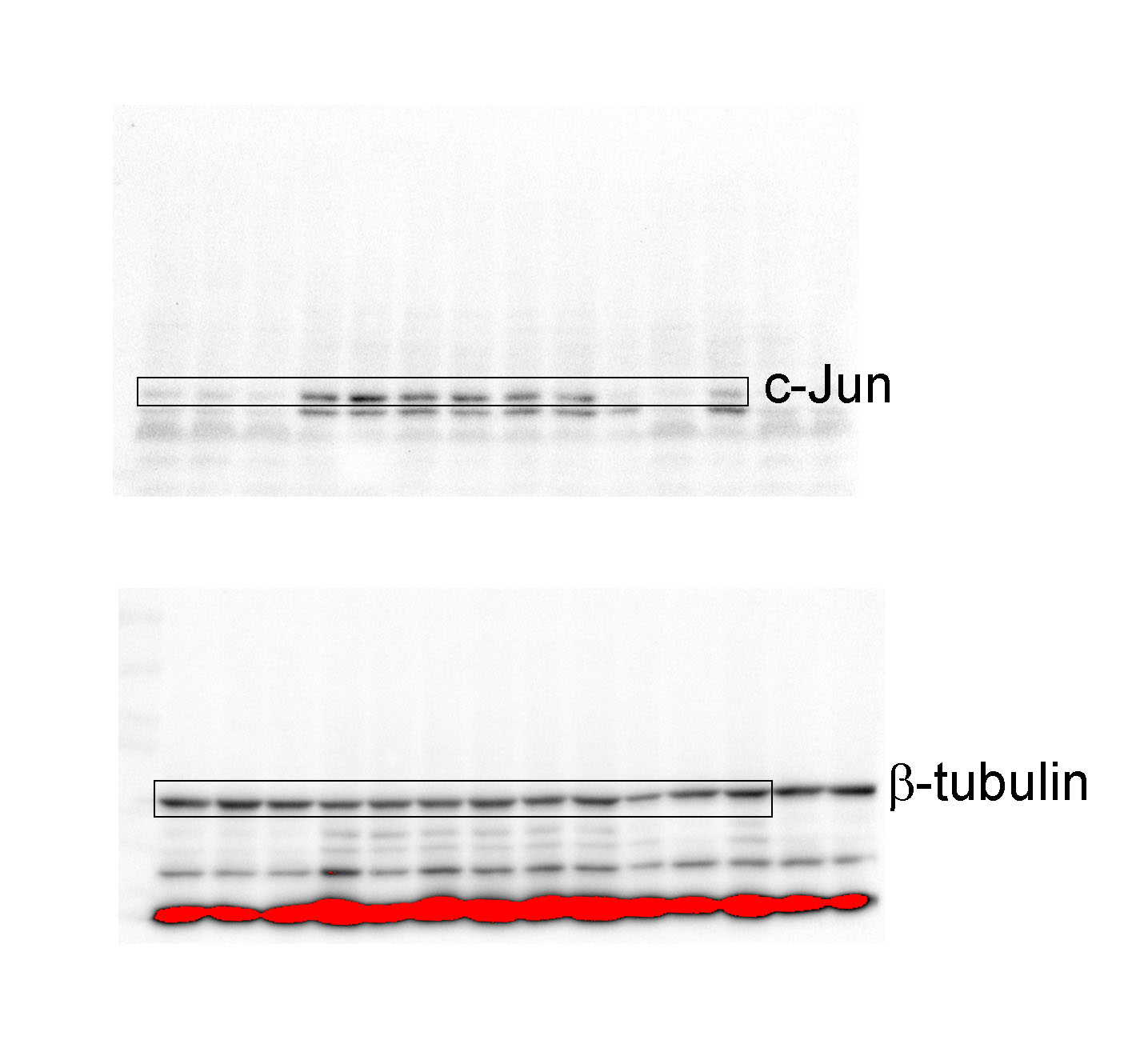

Supplement: Figure 6—source data 1. [file elife-66278-fig6-data1.zip › Figure 6 - source data 1/Annotated uncropped WB.jpg]

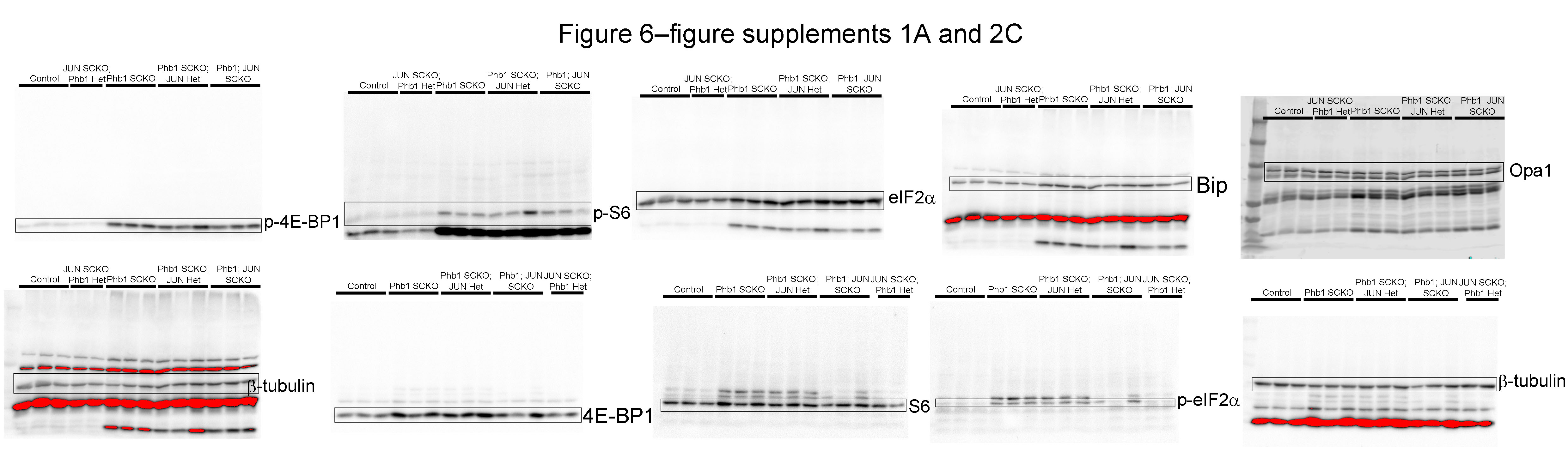

Supplement: Figure 6—figure supplement 1—source data 1. [file elife-66278-fig6-figsupp1-data1.zip › Figure 6 - figure supplement 1 - source data 1/Annotated uncropped WB.jpg]

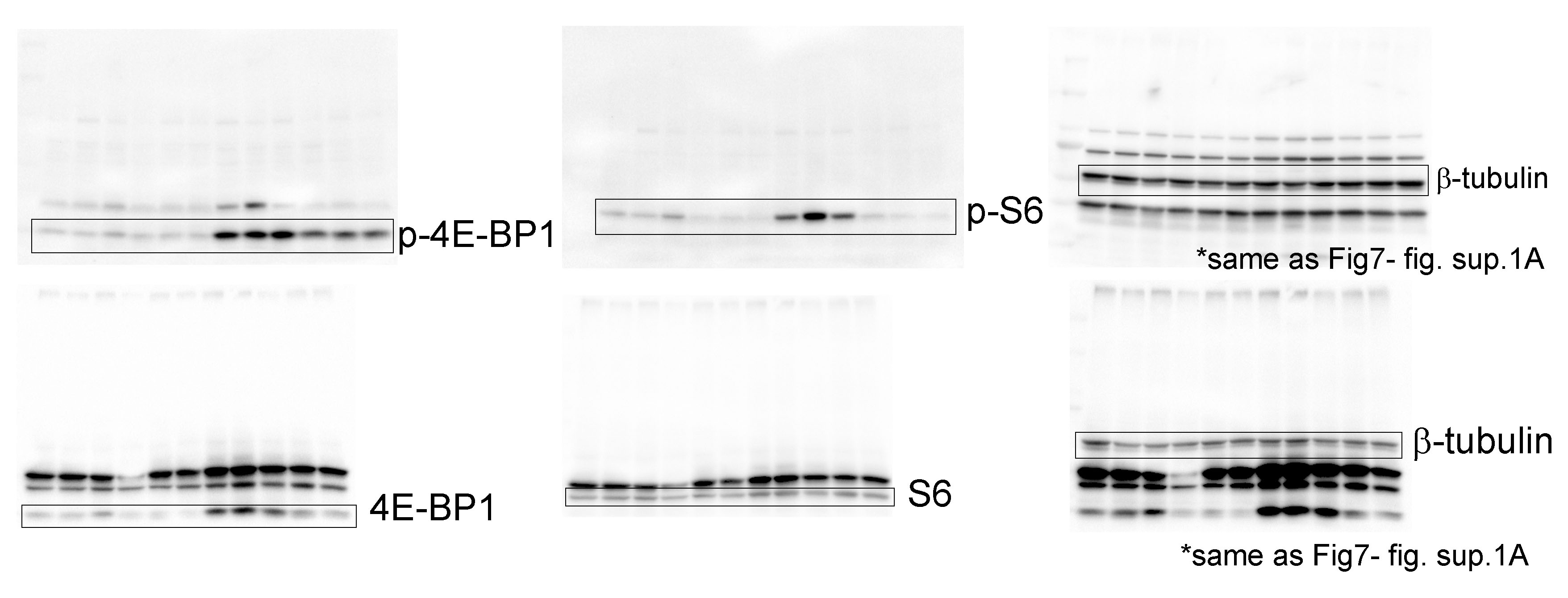

Supplement: Figure 7—source data 1. [file elife-66278-fig7-data1.zip › Figure 7 - source data 1/Annotated uncropped WB.jpg]

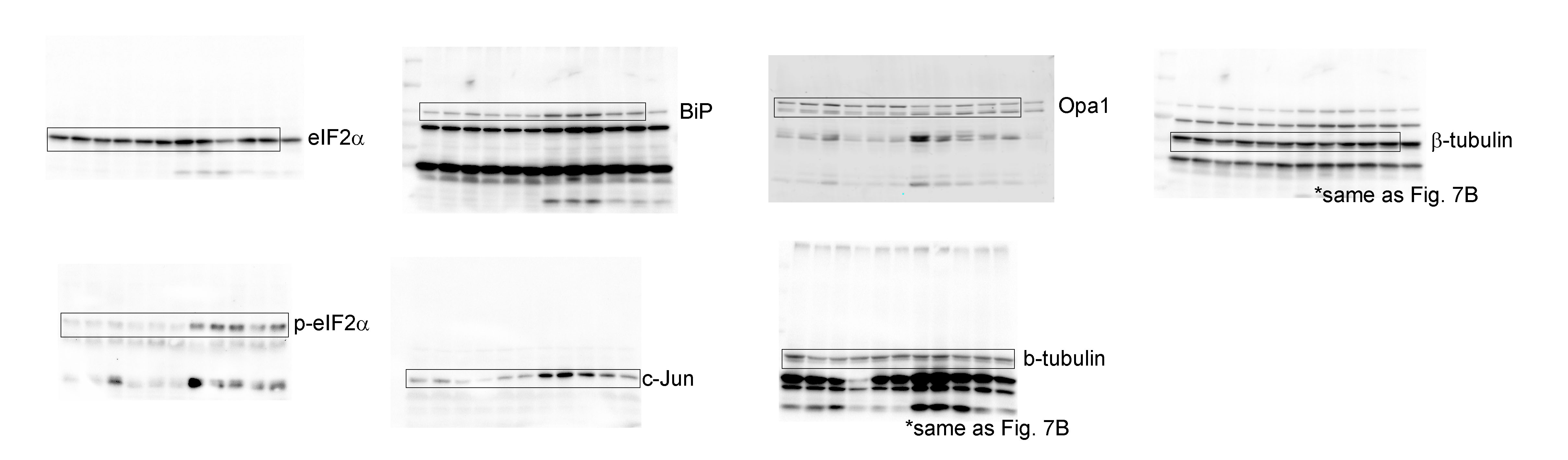

Supplement: Figure 7—figure supplement 3—source data 1. [file elife-66278-fig7-figsupp3-data1.zip › Figure 7 - figure supplement 3 - source data 1/Annotated uncropped WB.jpg]
